# Endopancreatic Bile Duct Cholangiocarcinoma in a Patient with Peutz-Jeghers Syndrome

**DOI:** 10.1155/2011/364570

**Published:** 2011-06-13

**Authors:** Alexandros K. Charalabopoulos, Sylvia P. Krivan, Nikolas A. Machairas, Evangelos P. Misiakos, Anastasios N. Machairas

**Affiliations:** 3rd Department of Surgery, Attikon University Hospital, University of Athens Medical School, 1 Rimini Street, Chaidari, 12462 Athens, Greece

## Abstract

Peutz-Jeghers syndrome is a rare autosomal dominant inherited disease characterized by a special type of hamartomatous gastrointestinal polyps combined with mucocutaneous melanin pigmentations. Patients with the syndrome have a high risk of developing neoplasia, with colon, small bowel, and stomach being the most common gastrointestinal sites. Herein, we present the occurrence of a rare tumor in patients with Peutz-Jeghers syndrome; a cholangiocarcinoma of the endopancreatic bile duct. A minireview is also presented. It can be concluded that cholangiocarcinoma remains a possible diagnosis in PJS patients, as in others that present with biliary obstruction. PJS patients may be at higher risk than others in view of their propensity for malignancy.

## 1. Case Presentation

A 31-year-old Caucasian male patient of Greek origin presented with a progressively increasing painless obstructive jaundice. The patient was known to have Peutz-Jeghers syndrome (PJS) since childhood. He was genetically tested for the syndrome (germline mutation in STK11), which was confirmed in Norwich, England where the patient was born and lived for 25 years. There was no family history of PJS or gastrointestinal carcinoma. Four months ago, the patient underwent surgery for intestinal obstruction and intussusception where a part of small bowel was excised. Biopsy revealed hamartomatous polyps confirming histological proof of PJS. The patient made a good recovery but 4 months later developed painless obstructive jaundice and was referred to our surgical department for further investigation and treatment.

Clinical examination confirmed the jaundice, and there were clear manifestations of PJS, with pigmented lesions around the mouth and on the hands. The patient reported weight loss in the last couple of months with no other apparent clinical abnormalities. A well-healed midline abdominal surgical scar was found. Biochemical investigations confirmed the obstructive nature of the jaundice with a direct bilirubin of 5.56 mg/dL, alkaline phosphatase of 231 U/L, and aspartate aminotransferase of 163 U/L. Haemoglobin was 10.4 gr/dL, and the prothrombin time was prolonged at 15.2 seconds. Tumor markers were negative. X-rays of the chest and abdomen were unremarkable.

**Figure 1:**
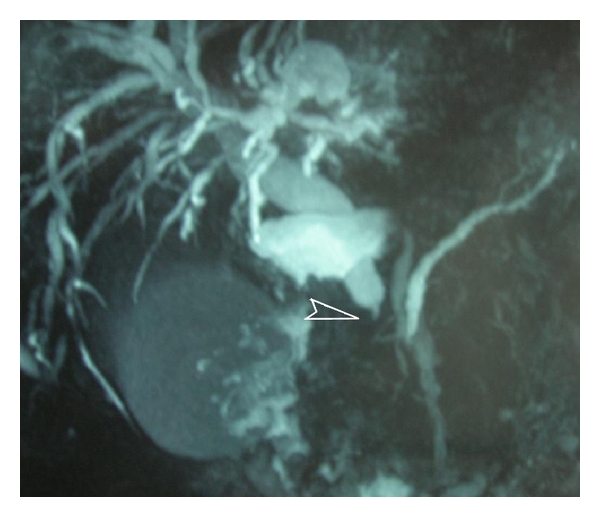
Magnetic resonance cholangiopancreatography showing dilated biliary tree and gallbladder. Arrow points at obstruction at the lower end of the common bile duct.

**Figure 2:**
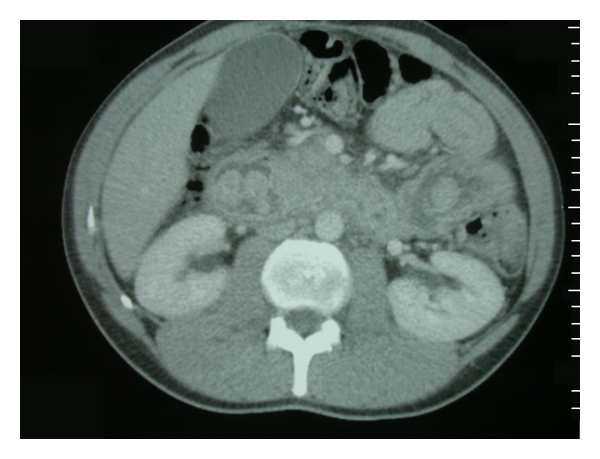
Contrast-enhanced axial computer tomography scan showing multiple polyps of the stomach, duodenum, and ileum.

Ultrasound showed dilated biliary tree and gallbladder. MRCP confirmed the previous findings and revealed an obstruction at the lower end of the common bile duct (Figure 1). This was confirmed by CT scan which also revealed multiple polyps of the stomach, duodenum, and intestine (Figure 2). ERCP was unsuccessful owing to difficulty revealing Vater's ampulla due to space-occupying duodenal polyps. 

**Figure 3:**
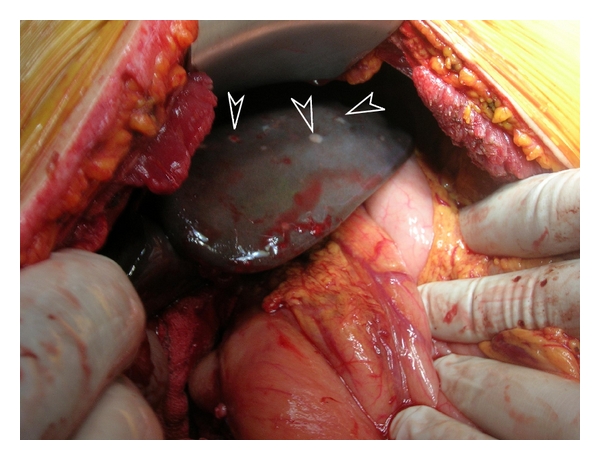
Intraoperative finding of diffuse rice-like metastases throughout the liver (arrows).

**Figure 4:**
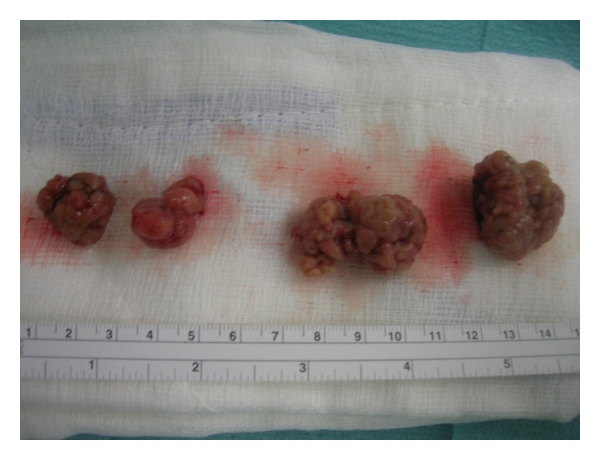
Excised large Peutz-Jeghers polyps from the duodenal wall.

A decision for the patient to undergo surgical laparotomy was taken. The duodenum was found distended and full of polyps. The gallbladder and the common bile duct were grossly dilated. The liver carried diffuse rice-like metastases throughout (Figure 3); enlarged hepatoduodenal lymph nodes were also identified. Frozen section biopsy of the liver and lymph nodes revealed metastasis from adenocarcinoma of unknown origin. The duodenum was dissected, and the polyps were excised (Figure 4). Due to the progression of the disease, cholecystectomy and end-to-side choledochojejunal anastomosis were performed. A 1 cm ring from the distal cut end of the bile duct was also sent for pathology examination.

The pathology report revealed a poorly differentiated cholangiocarcinoma of the common bile duct with liver metastases and positive hepatoduodenal ligament lymph nodes (station 12b). The carcinoma presented partially neuroendocrine differentiation. The immunophenotype of the neoplasm was the following: AE1/AE3:+++, CK7:+++, CAM5.2:+++, polyclonic CEA:++, CK34Be12:+, CK18:+, synaptophysin:+++, NSE:+, polyclonic Ca 19-9:−, Ki67:+ in 30% of cancer cells, P53:+ in 80% of cancer cells, Cerb2: expressed in <10%, EGFR:+ in 30%, and Ckit:−.

Postoperatively, the patient made an uneventful recovery and was discharged home two weeks later. A month postoperatively, he started medical treatment with chemotherapy. He received 3 cycles of oxaliplatin, capecitabine, and bevacizumab. Decision not to include another chemotherapeutic agent like mTOR inhibitors (e.g., everolimus) to the regimen was made by the surgical and medical oncology teams despite the partially neuroendocrine nature of the neoplasm. The patient showed no response to treatment and deceased 4 months later of generalized carcinomatosis.

## 2. Discussion

Peutz-Jeghers Syndrome (PJS) is a rare autosomal dominant inherited disease characterized by a special type of hamartomatous gastrointestinal polyps (PJ polyps) combined with mucocutaneous melanin pigmentations [1, 2]. PJ polyps are characterized by extensive smooth muscle arborization throughout the polyp and occur throughout the alimentary tract—sparing the esophagus—with a predilection for the small bowel with decreasing frequency from jejunum, ileum, colon, rectum, stomach, to duodenum. Extraintestinal polyps are rare but may occur in the ureter, nasal cavity, or gallbladder. Pigmented lesions are detectable in 95% of patients with PJS and are potentially disappearing with age. They vary in size, number, and color [3]. Predilection sites are the lips, the peri- and intraoral regions and less commonly the rectum, feet, vulva, and conjunctiva. The diagnosis of PJS is based on criteria including at least two PJ polyps, one PJ polyp and mucocutaneous pigmented lesions, or one PJ polyp and positive family history of PJS [4].

Negative family history (up to 45% of index cases) indicates a high incidence of de novo germline mutations. Symptoms, typically occuring in adolescence or young adulthood, include recurrent colicky abdominal pain due to intussusception of small bowel segments caused by large polyps and can be complicated by acute intestinal obstruction and paralytic ileus of a small bowel segment. Occult gastrointestinal bleeding and iron deficiency anemia may be other presentations of the syndrome. Impressively, pigmentation may be present in early childhood and may fade away or even disappear over the lifetime [3, 4]. 

Areas with intraepithelial neoplasia and cancer may occur in PJ polyps. Up to date, whether intraepithelial neoplasia predisposes to cancer following a “hamartoma-adenoma-carcinoma sequence” is still debatable. There are studies suggesting that a “hamartoma-adenocarcinoma sequence” can be applicable [5, 6]. On the other hand, and what is believed to be the case today for the majority of PJ polyps, is what has been shown from more recent studies. Based on the rarity of the atypia/dysplasia found on the PJ polyps, these studies argue against a “hamartoma-adenoma-carcinoma sequence” [7]. The clinical course is usually characterized by multiple surgical procedures for benign small bowel complications (intussusception, bleeding, and obstruction) [4]. 

The causative locus for PJS was elucidated by targeted linkage analysis and comparative genomic hybridization to be on chromosome 19p13-3 [8]. The gene that has been identified as LKB1 (also known as STK11) is ubiquitously expressed in all fetal and adult tissues and encodes for a serine-threonine protein kinase [9, 10]. It is involved in the regulation of cell proliferation and polarity acting as a tumor suppressor and also playing a crucial role in the regulation of adenosine-5-monophosphate-activated protein kinase energy homeostasis cascade [11, 12]. LKB1 mutants fail to activate glycogen synthase kinase (GSK) 3*β*, thus, preventing it from inhibiting the Wnt signaling pathway [13].

The dual phosphatase and tumor suppressor protein PTEN also interacts with the LKB1 protein. Several LKB1 point mutations associated with PJS disrupt the interaction with PTEN, suggesting that the loss of this interaction might contribute to PJS. Since PTEN is mutated in autosomal inherited disorders with phenotypes similar to those of PJS (Bannayan-Riley-Ruvalcaba syndrome and Cowden disease), there appears to be a functional link between the proteins involved in different hamartomatous polyposis syndromes, thus, emphasizing the central role played by LKB1 as a tumor suppressor in the intestine [14]. 

What current data is showing and what we nowadays are based on is that STK11/LKB1 mutations can be recognized in 84% and up to almost 100% of cases, depending on the patient selection criteria and the methods used for mutation search [7, 15]. In contrast, past studies of more than a decade ago reported ambiguous results such as 50% of PJS patients having no detectable germline mutation, families not linked to 19p13-3, and even a possible second PJS locus at 19p13-4 [16–19]. Genetic heterogeneity can thus be expected. A genotype-phenotype correlation has not been firmly established in PJS, probably owing to the small size of reported series. There are some initial data, however, suggesting that the absence of an LKB1 mutation carries a higher risk of cancer development, especially biliary carcinoma [20]. One study proposed that a mutation in exon 3 of the LKB1 gene carries a higher cancer risk than other LKB1 mutations [21]. Whereas clinical interfamilial variation might simply reflect different germline mutations, intrafamilial variation is also well documented. The variability includes the cancer risk, so better understanding should have a significant impact in forming individualized cancer surveillance/screening programs [22].

## 3. Conclusion

Patients with PJS have a high risk of developing cancer with it being around 85% by the age of 70 [23–25]. A large recent study including 419 PJS patients reported a risk for gastrointestinal cancers of 57% by the age of 70. Colorectal cancer was the most common gastrointestinal cancer with a lifetime risk of 39%, whereas the cumulative lifetime risk for pancreatic cancer was 11% [24]. 

The risk of small bowel, gastric, and biliary cancer is also increased. Extraintestinal cancers include gynecologic neoplasia (breast, ovary, and endometrium). The reported risk of developing breast cancer ranges from 31% to 50% [26]. Cancer is uncommon before the age of 30. The risk of cervical carcinomas is not increased, but female patients with PJS are at increased risk for the development of an adenoma malignum, which may show rapid disease progression and early dissemination. Almost all female patients with PJS develop an otherwise rare potentially malignant ovarian tumor, the sex cord tumor with annular tubules (SCTAT) [27]. Malignant transformation is observed in 20% of all cases. Cancers of the thyroid gland, gallbladder, and lung also seem to be associated with PJS. Sertoli cell tumors are considered to be the equivalent of SCTAT in male patients with PJS and causes gynecomastia. The risk of malignancy is estimated to be 10–20% [26, 28]. 

The well-recognized risk for patients with PJS to develop cancer has led to the orchestration of various screening programs. St. Mark's Hospital PJS surveillance program is the most widely applied and comprises of upper gastrointestinal endoscopy and colonoscopy every 3 years. Given the rarity of pancreatic cancer in this setting, no recommendations can be made on pancreatic screening in these patients. Nevertheless, as shown in our case, clinicians have to be aware that the endopancreatic portion of the bile duct can also be a location for PJS-associated cancer. Thus, the possibility of malignancy in any case of jaundice in this population should be taken into account before attributing the rising bilirubin to polyp-derived obstruction that is the most common but benign cause of jaundice in these patients.

The occurrence of cholangiocarcinoma of endo-pancreatic common bile duct in our young patient with PJS was characterized by the strikingly poor prognosis due to the inoperability of this case despite the immediate treatment from the time of presentation. Thus, further research is needed in order to elucidate the risk factors of this patient population to develop malignant transformations of their lesions. Proper risk stratification and closer followup in selected groups will promote well-timed diagnosis and proper intervention that may be life-saving.
